# Coupling Genetic Addiction Risk Score (GARS) with Electrotherapy: Fighting Iatrogenic Opioid Dependence

**DOI:** 10.4172/2155-6105.1000163

**Published:** 2013-10-31

**Authors:** Kenneth Blum, Marlene Oscar-Berman, Nicholas DiNubile, John Giordano, Eric R Braverman, Courtney E Truesdell, Debmalya Barh, Rajendra Badgaiyan

**Affiliations:** 1 Department of Psychiatry, McKnight Brain Institute, University of Florida, College of Medicine, Gainesville, FL, USA; 2 Departments of Psychiatry and Anatomy & Neurobiology, Boston University School of Medicine, and Boston, VA Healthcare System, Boston, MA, USA; 3 Department of Orthopaedic Surgery at the University of Pennsylvania, Philidelphia, Pennslyvania, USA; 4 Department of Holistic Medicine, National Institute for Holistic Addiction STudies, Inc. North Miami Beach, Fl, USA; 5 Department of Clinical Neurology, PATH Foundation NY, New York, NY, USA; 6 Center for Genomics and Applied Gene Technology, Institute of Integrative Omics and Applied Biotechnology (IIOAB), Nonakuri, Purbe Medinpur, West Bengal, India; 7 Department of Psychiatry, University at Buffalo & Harvard University, Psychiatrist, WNY VAMC, Buffalo, NY, USA; 8 Dominion Diagnostics, LLC., North Kingstown, Rhode Island, USA; 9 Department of Addiction Research & Therapy, Malibu Beach Recovery Center, Malibu Beach California, USA; 10 Department of Nutrigenomics, IGENE LLC., Austin Texas, USA

**Keywords:** Pain, Analgesia, Electrotherapy, Genetic addiction risk score, Dopamine, Substance use disorder (SUD)

## Abstract

The endemic of legal opioid iatrogenic induced prescription drug abuse is of major world-wide concern. Understanding pain pathways and the role of dopaminergic tone in the neurophysiology of pain relief provides potential therapeutic solutions. A 2011 NIDA report indicated that approximately 8.7% of the entire US population above the age of 12 years has used a psychoactive drug within the past 30 days. It has been reported that the overall genetic contribution to the variance of Substance Use Disorder (SUD) was approximately 60% but each candidate gene evaluated by GWAS was relatively small. In an attempt to combat this global endemic we are proposing a number of alternative strategies. Prevention of death due to opioid overdose and attenuation of prescription abuse should focus on strategies that target 1) high-dosage medical users; 2) persons who seek care from multiple doctors; 3) persons involved in “drug diversion”; 4) genetic testing for addiction liability and severity indices; 5) non-pharmacolgical analgesic treatments such as electrotherapy.

## A Legal Endemic in Pain Relief

**Figure 1:**
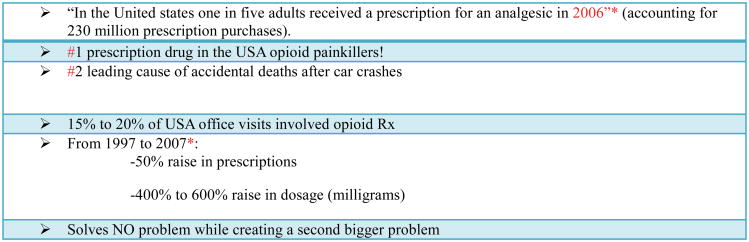
The Pain Game

The devastation continues in terms of prescription drug abuse. In 2007, approximately 27,000 unintentional (Iatrogenic) drug overdose deaths occurred in the United States, one death every 19 minutes [1,2]. Unfortunately, prescription drug abuse is the fastest growing addiction in the United States. The increase in iatrogenic drug overdose death rates has been driven by increased use of a class of prescription drugs called opioid analgesics. Since 2003, more overdose deaths have involved opioid analgesics than even heroin and cocaine combined [3,4]. For every unintentional overdose death related to an opioid analgesic, 9 individuals are admitted for substance use disorder (SUD) treatment, 35 visit emergency departments, 161 report drug abuse or dependence, and 461 report nonmedical uses of opioid analgesics [5] (Figure 1).

Patients with a history of drug or alcohol addiction are known to present to physicians with pain complaints. There is a paucity of the medical literature on the treatment of pain with opioids in patients in recovery or active addiction due to inconsistent criteria. There are clear differences between physical dependence, tolerance, and addiction. Addiction is different from pseudo addiction especially regarding physical dependence, tolerance and must be determined by the patient's behavior after appropriate pain management. Other than opioids (long and short term) there are many other medications that can enhance pain control as adjunctive analgesics. Importantly, drug-seeking behavior may be seen with either active addiction or pseudo addiction, or as part of deviant behavior such as “drug diversion”. Safe prescribing of medications with abuse potential must be carefully monitored and genetic risk should be part of a prescreening in the pain field to reduce iatrogenic pseudo addiction.

Overall, rates of opioid analgesic misuse and overdose death are highest among men, aged 20-64 years, non-Hispanic whites, and poor and rural populations. Individuals who have mental illness are overrepresented among both those who are prescribed opioids and those who overdose on them. The two main populations in the United States at risk for prescription drug overdose are the approximately 9 million individuals who report long-term medical use of opioids, and about 5 million individuals who report nonmedical use (i.e., use without a prescription or medical need), in the past month [6].

## Problem of Prescription Opiates

In the past 15 years, the amount of opiates prescribed to treat chronic pain has increased significantly. Drug distribution through the pharmaceutical supply chain was the equivalent of 96 mg of morphine per person in 1997 and about 700 mg per person in 2007, an increase of >600% [6]. That 700 mg of morphine per individual is enough for everyone in the United States to take a typical 5 mg dose of Vicodin (hydrocodone and acetaminophen) every 4 hours for 3 weeks. Patients who abuse opioids have learned to exploit this increased practitioner sensitivity to pain, and clinicians struggle to treat patients without overprescribing these drugs.

Among patients who are prescribed opioids, an estimated 80% are prescribed relatively low doses (<100 mg morphine equivalent dose per day, the maximum recommended daily dose of vicodin is 60 mg BID) by a single clinician and these patients account for an estimated 20% of all prescription drug overdoses. Another 10% of patients are prescribed high doses (≥ 100 mg morphine equivalent dose per day) of opioids by single physicians and account for an estimated 40% of prescription opioid overdoses. Importantly, the remaining 10% of patients are of greatest concern. These are patients who seek care from multiple clinicians and are prescribed high daily doses, and account for another 40% of opioid overdoses. Patients in this third group not only are at high risk for overdose themselves but are likely diverting or providing drugs to others who are using them without prescriptions [7].

## Paving the Way to Overcoming an Unwanted Addiction Nightmare

Understanding the enormous endemic problem of prescription opioid induced Iatrogenic opioid dependence in pain patients provoked this Trieste on alternative procedures to potentially reduce risk and improve outcomes. This commentary will explore a paradigm shift in electrotherapeutic treatment used to augment tissue healing associated with human neuropathies and injuries. It is well known that besides a number of side effects reported for chronic opioid therapy, selective genetically predisposed individuals at risk for opioid dependence should be identified early in treatment [8,9].

## Problems with Opiate Treatment of Chronic Pain

As mentioned above, opiates are the most commonly prescribed medication for both acute and chronic pain. Usually short term (1-2 week) use of therapeutic dose of these drugs does not lead to long-term abuse in patients with no prior addiction history or genetic vulnerability to addiction. The abuse potential increases with increasing length and dose of opiate use. Many patients receive these medications for the conditions that last for several years and decades. These conditions include low back pain and trigeminal and other forms of neuralgia and neuropathies. In these patients opioid agents start losing analgesic efficacy because of development of tolerance. It requires them to increase the dose to achieve the same level of analgesia until the dose cannot be increased due to respiratory depression and other adverse effects. It results in inadequate pain control, which is made worse by opiate induced increased pain sensitivity. This alone puts the patients and clinicians in a bind. The problem however gets worse because of addiction. Thus, chronic users of opiates not only have inadequate pain control; they also become dependent on these drugs. Because of dependency they experience withdrawal symptoms (both physical and mental) attenuate reduce pain sensitivity is contemplated. Many patients in this situation overdose. The overdose is not merely an attempt to alleviate pain and frustration but also to act on active or passive suicidal ideations, particularly because opiates are also known to cause depression leading to suicidality.

Opiates therefore are not the safe treatment for chronic pain. There is need to explore alternate strategies. These strategies can take advantage of our understanding of the brain mechanisms of pain control and pain perception. Even though understanding of these mechanisms is limited at this time, there is enough information to develop non-pharmacological techniques of controlling chronic pain. These techniques are particularly important for individuals with greater genetic vulnerability to opiate dependence.

A number of electrotherapeutic treatment methods have been developed to treat chronic pain. These methods have varying degrees of success in treating chronic pain. We are proposing a novel non-pharmacological approach, which involves coupling of genetic information and modulation of pain mechanisms through electrotherapy.

## The New Deal: GARS and H-Wave

A number of genes and associated polymorphism are believed to impact pain tolerance and sensitivity [10]. Thus, identification of candidate gene polymorphism provides a unique therapeutic target to assist in pain treatment. We propose that pharmacogenetic testing of candidate genes (i.e., mu receptors, PENK etc.) will result in pharmacogenomic solutions personalized to the individual patient, with potential improvement in clinical outcome [11] especially in those patients who carry risk alleles as identified by the Genetic Addiction Risk Score (GARS) test (IGENE, Inc. & Dominion Diagnostics, Austin, TX). The test identifies alleles known to impart vulnerability to addiction and makes an assessment of the degree of vulnerability of an individual to develop addictive behavior. It also predicts the severity of addiction in an individual. This information could help in individualized selection of the type and duration of a non-pharmacological therapy at this time and in future it could be used to formulate gene therapy.

The non-pharmacological techniques modulate activity of pain pathway. They modulate the activity at different levels of ascending and descending pain pathways. While it is well established that the principal ascending pathways originate in the dorsal horn of the spinal cord and in the medulla, additional brain areas are involved in the control of sensitivity to pain. The most important being the descending pain pathways that originate from a number of brain areas including the anterior cingulate, periaqueductal grey, and hypothalamus.

A novel electrotherapeutic approach to modulate neural signals of pain involves manipulation of blood flow to effected area involving muscle stimulation and loading using a H-Wave device (Electronic Waveform Lab, Inc., Huntington Beach, CA). The H-wave device is a small diameter fiber stimulator, which uses a relatively high frequency wave form for analgesia and an ultra low frequency (2 Hz) waveform to stimulate low tension, non-tetanizing contraction, which mimics voluntary muscle contractions. The H-Wave Device Stimulation (HWDS) has been shown to reduce edema by stimulating smooth muscle fibers within the lymphatic vessels. Moreover, it induces nitric oxide (NO)-dependent augmentation of microcirculation and angiogenesis leading to accelerated tissue healing [12-15]. It accelerates healing also by inducing small muscle contraction. HWDS does not stimulate the motor nerves of large white muscle fibers or the sensory delta and C pain nerve fibers. Thus, the painful effects of tetanizing fatigue, which reduces trans-capillary fluid shifts, are eliminated and healing is accelerated [12].

Thus, HWDS attenuates pain by delivering relatively high frequency wave forms and also by accelerating healing by delivering ultra- low frequency wave forms. A recent meta-analysis found a moderate-to-strong positive effect of HWDS in providing pain relief (reducing or eliminating the need for pain medication) and in enhancing functionality [16].

**Figure 2:**
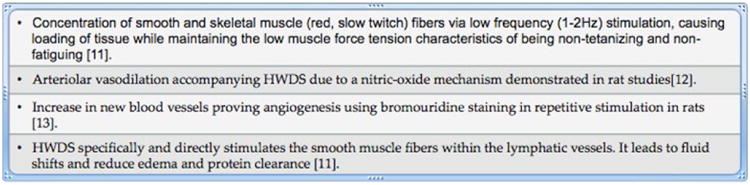
Characteristics of H-Wave Electrotherapy

A number of physiological mechanisms of action of HWDS have been investigated in animals. Figure 2 illustrates the mechanism, which is responsible for improvements in tissue circulation.

## Summary

Prescription drug abuse is becoming endemic across the United States and several European countries, as the proportion of people seeking treatment for prescription drug addiction continue to increase. In the US, opioids, tranquilizers, sedatives and stimulants are the leading drugs that are abused. The number of people seeking treatment help for opioid addiction increased more than 400%-600% in the decade ending in 2008. Currently Pseudo-addiction it is still a major health problem globally. However, only one in a hundred people abusing prescription opioids get treatment [17].

Prevention of death due to opioid overdose and attenuation of prescription abuse should focus on strategies that target 1) high-dosage medical users and 2); persons who seek care from multiple doctors; 3) these persons are likely involved in drug diversion; 4) genetic testing for addiction liability [18-21] and severity indices [22] and 5) non-pharmacological analgesic treatments such as electrotherapy [23].

## References

[1] Zhang H, Kranzler HR, Yang BZ, Luo X, Gelernter J (2008). The OPRD1 and OPRK1 loci in alcohol or drug dependence: OPRD1 variation modulates substance dependence risk. Mol Psychiatry.

[2] CDC (2011). Policy impact: prescription painkiller overdoses.

[3] Hall AJ, Logan JE, Toblin RL, Kaplan JA, Kraner JC (2008). Patterns of abuse among unintentional pharmaceutical overdose fatalities. JAMA.

[4] Black RA, Trudeau KJ, Cassidy TA, Budman SH, Butler SF (2013). Associations between public health indicators and injecting prescription opioids by prescription opioid abusers in substance abuse treatment. J Opioid Manag.

[5] Centers for Disease Control and Prevention (CDC) (2012). CDC grand rounds: prescription drug overdoses - a U.S. epidemic. MMWR Morb Mortal Wkly Rep.

[6] Lynch M (2013). Nonmedical use of prescription opioids: what is the real problem?. Pain Res Manag.

[7] Rice JB, White AG, Birnbaum HG, Schiller M, Brown DA (2012). A model to identify patients at risk for prescription opioid abuse, dependence, and misuse. Pain Med.

[8] Michna E, Ross EL, Hynes WL, Nedeljkovic SS, Soumekh S (2004). Predicting aberrant drug behavior in patients treated for chronic pain: importance of abuse history. J Pain Symptom Manage.

[9] Dobrinas M, Crettol S, Oneda B, Lahyani R, Rotger M (2013). Contribution of CYP2B6 alleles in explaining extreme (S)-methadone plasma levels: a CYP2B6 gene resequencing study. Pharmacogenet Genomics.

[10] Chen AL, Chen TJ, Waite RL, Reinking J, Blum K (2009). Hypothesizing that brain reward circuitry genes are genetic antecedents of pain sensitivity and critical diagnostic and pharmacogenomic treatment targets for chronic pain conditions. Med Hypotheses.

[11] Blum K, Chen TJ, Meshkin B, Downs BW, Gordon CA Genotrim, a DNA-customized nutrigenomic product, targets genetic factors of obesity: hypothesizing a dopamine-glucose correlation demonstrating reward deficiency syndrome (RDS). Med Hypotheses.

[12] Blum K, Ho CK, Chen AL, Fulton M, Fulton B (2008). The H-Wave((R)) Device Induces NODependent Augmented Microcirculation and Angiogenesis, Providing Both Analgesia and Tissue Healing in Sports Injuries. Phys Sportsmed.

[13] Smith TL, Callahan MF, Blum K, Dinubile NA, Chen TJ (2011). H-Wave® effects on blood flow and angiogenesis in longitudinal studies in rats. J Surg Orthop Adv.

[14] Smith TL, Blum K, Callahan MF, DiNubile NA, Chen TJ (2009). H-Wave induces arteriolar vasodilation in rat striated muscle via nitric oxide-mediated mechanisms. J Orthop Res.

[15] Blum K, DiNubile NA, Tekten T, Chen TJ, Waite RL (2006). H-Wave, a nonpharmacologic alternative for the treatment of patients with chronic soft tissue inflammation and neuropathic pain: a preliminary statistical outcome study. Adv Ther.

[16] Blum K, Chen AL, Chen TJ, Prihoda TJ, Schoolfield J (2008). The H-Wave device is an effective and safe non-pharmacological analgesic for chronic pain: a meta-analysis. Adv Ther.

[17] Chen TJ, Blum K, Chen AL, Bowirrat A, Downs WB (2011). Neurogenetics and clinical evidence for the putative activation of the brain reward circuitry by a neuroadaptagen: proposing an addiction candidate gene panel map. J Psychoactive Drugs.

[18] Blum K, Oscar-Berman M, Giordano J, Downs B, Simpatico T (2012). Neurogenetic Impairments of Brain Reward Circuitry Links to Reward Deficiency Syndrome (RDS): Potential Nutrigenomic Induced Dopaminergic Activation. J Genet Syndr Gene Ther.

[19] Fonseca F, de la Torre R, Díaz L, Pastor A, Cuyàs E (2011). Contribution of cytochrome P450 and ABCB1 genetic variability on methadone pharmacokinetics, dose requirements, and response. PLoS One.

[20] Blum K, Gardner E, Oscar-Berman M, Gold M (2012). “Liking” and “wanting” linked to Reward Deficiency Syndrome (RDS): hypothesizing differential responsivity in brain reward circuitry. Curr Pharm Des.

[21] Butler SF, Fernandez K, Benoit C, Budman SH, Jamison RN (2008). Validation of the revised Screener and Opioid Assessment for Patients with Pain (SOAPP-R). J Pain.

[22] Blum K, Chen TJ, Ross BD (2005). Innate properties of H-Wave device, a small fiber stimulator provides the basis for a paradigm shift of electro-therapeutic treatment of pain with increased functional restoration associated with human neuropathies by affecting tissue circulation: a hypothesis. Med Hypotheses.

[23] Weaver MF, Schnoll SH (2002). Opioid treatment of chronic pain in patients with addiction. J Pain Palliat Care Pharmacother.

